# Ipilimumab treatment decreases circulating Tregs and GrMDSC while enhancing CD4+ T cell activation

**DOI:** 10.1186/1479-5876-13-S1-O7

**Published:** 2015-01-15

**Authors:** Yago Pico de Coaña, Maria Wolodarski, Giusy Gentilcore, Yuya Yoshimoto, Isabel Poschke, Johan Hansson, Giuseppe V Masucci, Rolf Kiessling

**Affiliations:** 1 Department of Oncology-Pathology, Cancer Center Karolinska, Karolinska Institutet, Stockholm, Sweden; 2 Division of Molecular Oncology of Gastrointestinal Tumors, German Cancer Research Center, Heidelberg, Germany

## Background

Ipilimumab is a fully human antibody that blocks CTLA-4 and has proven to extend overall survival in patients with unresectable stage III or stage IV melanoma. There is a need for well-documented pharmacodynamic markers together with potential predictive biomarkers that may allow for pretreatment selection of patients and screening for IRAE. Most of the recently published immune monitoring studies focus mainly on the effect that ipilimumab has on T cell populations. An in-depth immune monitoring study was conducted in advanced melanoma patients undergoing treatment with ipilimumab. The main focus of this work was to analyze the effect of ipilimumab treatment on peripheral blood MDSC populations as well as T cells.

## Materials and methods

Six patients with advanced stage melanoma received ipilimumab treatment at 3 or 10 mg/kg doses as part of double blind randomized trial CA184-169. Twenty-four additional patients received 3 mg/kg doses. Blood samples were collected before treatment (baseline) and at the time of the second and fourth ipilimumab doses. Peripheral blood mononuclear cells were isolated by density gradient centrifugation and stained for flow cytometric analysis within two hours of sample collection.

## Results

In the thirty patients included, median OS was 38 weeks from the start of treatment. Adverse events were observed in 12 patients (40%), including seven grade III-IV (23%). Patients were classified according to their response as PD (progressive disease, 57%), SD (stable disease, 23%) and PR (partial response, 20%).

ICOS^+^CD4^+^ T cell frequency was significantly increased after Ipilimumab treatment, suggesting an increase in the activation of this cellular population. The endpoint mean frequency of Tregs was significantly lower than the baseline. In addition to this, changes in the suppressive myeloid compartment were also observed, with significant reductions in the frequency of granulocytic MDSCs. The suppressive potential of both granulocytic and monocytic MDSCs was also diminished, as the frequencies of Arg1^+^ and iNOS^+^ cells were reduced after treatment. Further analysis showed that the populations of granulocytic MDSCs and CD4^+^ICOS^+^ T cell populations presented a statistically significant correlation.

## Conclusions

Ipilimumab treatment can be acting in two very distinct ways: on one hand it is increasing the activation status of T cells and on the other hand, it is decreasing the frequencies of suppressive cells such as Tregs and granulocytic MDSCs. These effects, and their possible correlations to clinical benefit, should be further explored.

